# Efficacy of High-Dose Vitamin C Infusion on Outcomes in Sepsis Requiring Mechanical Ventilation: A Double-Blind Randomized Controlled Trial

**DOI:** 10.1155/2022/4057215

**Published:** 2022-07-15

**Authors:** Wessam A. El Driny, Ibrahim M. Esmat, Sara M. Shaheen, Nagwa A. Sabri

**Affiliations:** ^1^Ain-Shams Specialized Hospital, Cairo, Egypt; ^2^Department of Anesthesia and Intensive Care, Faculty of Medicine, Ain-Shams University, Cairo, Egypt; ^3^Department of Clinical Pharmacy, Faculty of Pharmacy, Ain-Shams University, Cairo, Egypt

## Abstract

**Background:**

Critically ill patients have an increased requirement for vitamin C in sepsis and these patients have low levels of vitamin C. The researchers validated the efficacy of high-dose vitamin C intravenous infusion (IVI) in patients with sepsis requiring mechanical ventilation.

**Methods:**

Forty patients were randomly assigned to 2 groups (20 each) in a 1 : 1 ratio in accordance with the vitamin C treatment regimen: Group I (GI): patients received 1.5 g/6 h vitamin C in 50 ml of dextrose 5% in water (D5W) IVI over 30 minutes for 4 consecutive days; Group II (GII): patients received 100 mg vitamin C/day as a first single dose in 50 ml of D5W IVI over 30 minutes and the other three subsequent doses were 50 ml of plain D5W IVI over 30 minutes for 4 consecutive days. Primary outcomes were the change in sequential organ failure assessment (SOFA) score at day 7, the incidence of ventilator-associated pneumonia (VAP), and the plasma vitamin C level. The glutathione peroxidase (GPX) activity, C-reactive protein (CRP) level, duration of vasopressor therapy, and 28-day mortality were secondary outcomes.

**Results:**

The change in SOFA score at day 7 showed a significant difference between GI and GII (*p* < 0.001). The incidence of early VAP was significantly lower in GI (*p*=0.044). Vitamin C levels showed a significant rise in GI at day 1 and day 4 (*p* < 0.001 and *p* < 0.001, respectively). GPX activity of day 4 and day 7 was significantly higher in GI (*p*=0.005 and *p*=0.014, respectively). CRP levels of day 4 and day 7 were significantly higher in GII (*p* < 0.001 and *p* < 0.001, respectively). There was a significant difference in 28-day mortality (*p*=0.038) and duration of vasopressor therapy (*p*=0.033) in GI compared to GII.

**Conclusion:**

The early use of high-dose vitamin C intravenous infusion in patients with sepsis requiring mechanical ventilation in combination with the standard treatment for sepsis lowered the incidence of VAP, increased the antioxidant status, and improved the illness severity. *Trial Registration.* This trial is registered with ClinicalTrials.gov Identifier (NCT04029675).

## 1. Background

Sepsis is known as the main cause of death in critically ill patients and is responsible for one-third to half of the hospital mortalities [1]. Sepsis also affects approximately 15–19 million annual cases worldwide, and most of them occur in developing countries [2]. Sepsis is caused by an infectious organism, especially Gram-positive infections, which triggers a local immune response [3]. The migration of neutrophils into the inflamed tissue will follow causing a release of free radicals and inflammatory mediators which may trigger, in some patients, a systemic inflammatory response syndrome (SIRS). SIRS may progress to severe sepsis, septic shock, and multiple organ dysfunction syndrome (MODS) [4]. Patients with sepsis may develop acute lung injury that is characterized by activation of tissue inflammation and reactive oxygen species (ROS) production [4].

Ventilator-associated pneumonia (VAP) is defined as pneumonia that develops 48–72 h following endotracheal intubation. VAP is a serious complication affecting approximately 30% of susceptible patients, so hospital length of stay (LOS), inpatient costs, and hospital mortality will increase. The mortality rate for VAP could reach rates up to 76% in severe lung infection [5]. Consequently, newer effective therapies to prevent the inflammation and decrease the incidence of VAP are necessary to improve patients' quality of care.

Vitamin C is a potent antioxidant, anti-inflammatory, and immune-support food supplement. It acts as an enzyme cofactor for the synthesis of collagen, cortisol, vasopressin, and catecholamines needed for the survival of patients with sepsis [6]. Vitamin C could attenuate sepsis-associated lung injury by preventing sepsis-induced cytokine storms and neutrophil accumulation [7]. Plasma vitamin C concentrations with scurvy levels (i.e., <23 *µ*mol/l) found in burn sepsis and other conditions with systemic inflammation and oxidative stress (OS) [8]. Many new clinical studies devoted during the COVID-19 outbreak reported that high-dose vitamin C decreased the mortality and improved oxygen support status in coronavirus cases without adverse events [9, 10]. Patients with sepsis may require significantly higher intakes of vitamin C due to the high depletion during the severe inflammatory response [8]. Parenteral vitamin C nutrition is preferable to enteral one because its intravenous intake bypasses the rate-limiting intestinal uptake of oral dose [8].

Glutathione (GSH), an important intracellular antioxidant, is converted to its oxidized form (GSSG) by glutathione peroxidase (GPX). When glutathione is used as a scavenger, it is converted back to its reduced form by glutathione reductase (GR). Daily oral supplementation with vitamin C increases the level of GPX and GR. These enzymes scavenge free radicals and hinder oxidative damage [11].

The investigators of this study validated the efficiency and safety of high-dose vitamin C intravenous infusion (IVI) to reduce the incidence of VAP in patients with sepsis requiring mechanical ventilation (MV).

## 2. Methods

### 2.1. Ethics

This study was registered at ClinicalTrials.gov (NCT04029675) after approval by the clinical pharmacy department of the Faculty of Pharmacy of Ain-Shams University, Cairo, Egypt (no. 170/2017). A written consent was obtained from each patient or his/her legal guardians.

### 2.2. Patients

This study was carried out on 40 adult patients admitted to the internal medicine intensive care unit (ICU) of Ain-Shams University Hospitals, from 1st of August 2019 to 15th of February 2020, aged between 18 and 70 years who met the criteria for sepsis based on the Surviving Sepsis Campaign [12] and required MV within 24 hours from ICU admission.

Exclusion criteria were age <18 years, body mass index (BMI) > 40, pregnant or breastfeeding, no informed consent from their relatives, moribund and not expected to survive 96 hours, pneumonia developed <48 hours following intubation, cancer as the cause of SIRS or sepsis, ischemic reperfusion injury, and chronic kidney diseases [13]. Patients were also ruled out if they had a history of aspiration before intubation, respiratory distress syndrome, or known allergy to vitamin C.

### 2.3. Randomization and Blinding

This study had a randomized and a double-blind design. The randomization of patients within the first 24 hours of ICU admission was carried out through computer-generated random numbers hidden in sealed opaque envelopes, and a ward nurse chose the envelope that determined the patient's group [14].

Forty patients were randomly assigned to 2 groups (20 each) in a 1 : 1 ratio in accordance with the vitamin C treatment regimen: Group I (GI): patients received 1.5 g/6 h vitamin C in 50 ml of dextrose 5% in water (D5W) IVI over 30 minutes for 4 consecutive days; Group II (GII): patients received standard daily requirements of vitamin C (100 mg/day) [15] as a first single dose in 50 ml of D5W IVI over 30 minutes and the other three subsequent doses were 50 ml of plain D5W IVI over 30 minutes for 4 consecutive days.

For blinding, the vitamin C dose was prepared by the ICU pharmacist and handled to the ICU nurse who was blinded to the solution composition. ICU residents responsible for data collection of this clinical trial were blinded to the assignment of patients to treatment groups [16].

The Dose 1 of the study drug was initiated within 2 hours after the randomization in both groups. The study drug infusion was stopped when the last dose (Dose 16) was administered or at ICU discharge, study withdrawal, or death, which of which happened first. The intervention drug was supplied as Cevarol® vitamin C 500 mg/5 ml by Memphis Pharmaceuticals and Chemical Industries-A.R.E.

Standard treatments for sepsis were given according to the surviving sepsis campaign guidelines [12]. According to the local institutional protocol of ICU, standard prophylactic measures for stress-related mucosal damage (SRMD), glycemic control (blood glucose level <150 mg/dL), and deep vein thrombosis (DVT) with either enoxaparin or heparin in patients (creatinine clearance <30 mL/min) were implemented within 24 hours from ICU admission. Early administration of broad-spectrum antibiotic therapy was also accomplished.

### 2.4. Ventilator Procedures and Fluid Administration

The MV was initiated with the Acute Respiratory Distress Syndrome Network (ARDSNet) tidal volume settings [17] and according to local institutional protocol. Ventilation modes; bilevel positive airway pressure (BiPAP) or synchronized intermittent mandatory ventilation (SIMV) + pressure support (PS), were allowed targeting delivering the prescribed tidal volume (VT, 6 ml/kg) of predicted body weight [17]. The oxygenation target was [55 mm Hg < partial pressure of oxygen (PaO_2_) < 80 mmHg] or [88% < oxygen saturation (SpO_2_) < 95%], and the minimal positive end-expired pressure (PEEP) was 5 cm H_2_O. Bicarbonate was given if pH < 7.30. The weaning readiness criteria [16] for every ventilated patient (invasively or noninvasively) were assessed every day between 6 and 10 AM. Different approaches were used if the weaning readiness criteria were met to accomplish spontaneous breathing trial procedure and assessment for unassisted breathing [17]. A decision to remove ventilatory support was made if tolerance criteria for spontaneous breathing trial were met for at least 30 minutes [17].

VAP was diagnosed and evaluated as either early VAP < 5 days or late VAP ≥ 5 days of MV [18]. The researchers excluded any persisting incidences of hospital-acquired pneumonia (HAP).

A conservative fluid administration protocol was started within the first 2 hours of randomization of patients to keep the mean arterial pressure (MAP) ranging between 60 and 90 mmHg with central venous pressure (CVP) in the range of 8–12 mmHg and urine output (UOP) of >0.5 ml/kg/h [19] with the limited use of sedative agents (e.g., dexmedetomidine) [20].

### 2.5. Outcomes

The following data were documented: demographic data, Acute Physiology and Chronic Health Evaluation II (APACHE II) score [21], comorbidities, diagnosis, type of infection according to culture results, and sequential organ failure assessment (SOFA) score [22]. Patients were followed up to 28 days or till death, which of which happened first.

The assessment of the following variables was done at days 0, 1, 4, and 7 after randomization: vital signs, hematological, biochemical data analysis, (PaO_2_/FiO_2_) ratio, SOFA score, plasma vitamin C level, GPX activity, and plasma C-reactive protein (CRP) level. Cultures of specimens drawn from any new site of infection after randomization were done when requested. The incidence of VAP, ventilator-free days (VFDs), duration of vasopressor support, length of ICU stay, and 28-day mortality were also assessed. Any vitamin C-related adverse effects were recorded. Day 0 meant that randomization was done in the ICU and treatment was immediately initiated.

The clinical assessment of patients with sepsis requiring MV was accomplished with the APACHE II score and SOFA score. In addition to the laboratory assessment with arterial blood gases, CRP level test which is a nonspecific inflammatory mediator and GPX activity which is a specific oxidative stress marker were recorded [18].

Primary outcomes of this study were the change in SOFA score at day 7, the incidence of VAP, and the plasma vitamin C level. The GPX activity, plasma CRP level, 28-day mortality, duration of vasopressor support, VFDs, and length of ICU stay were secondary outcomes.

### 2.6. Laboratory Analysis

All the laboratory blood samples (5 ml each) were drawn every day at 7 am from arterial lines and central venous lines. The first sample (day 0) was collected upon diagnosis of sepsis and prior to the beginning of the study. Other samples were collected on days 1, 4, and 7 after randomization. The levels of vitamin C and GPX were analyzed using automated ELISA kits (Human Vitamin C and Human Glutathione Peroxidase, Sinogeneclon Biotech Co., Ltd., China). The CRP was evaluated by a high-sensitivity C-reactive protein (hsCRP) assay using the hsCRP Diazyme kits (DZ135A-K hsCRP Test Kit) in accordance with manufacturer's recommendations on a Beckman au480 automated chemistry analyzer at Ain-Shams University Hospitals Laboratories, Cairo, Egypt.

All other routine laboratory tests were performed at the hospital laboratory based on clinicians' requests.

### 2.7. Statistical Analysis

#### 2.7.1. Power of the Study

Based on a previous study, the change in SOFA score was 7.8 ± 3.8 in the intervention group, while it was 4.7 ± 2.0 in the control group [18], assuming that the power 80% and the alpha error 5% and by using PASS 11^th^ release program [23], the minimum sample size was 17 patients per group. The research team enrolled 20 cases per group for possible attrition.

#### 2.7.2. Data Analysis

IBM Statistical Package for Social Sciences (SPSS) version 22.0, IBM Corp., Chicago, USA, 2013 was used for data management and analysis. Quantitative data were described using mean ± SD if normally distributed and median (1^st^–3^rd^ Inter-quartiles) if not normally distributed. Normality was tested using Shapiro–Wilk test. Number and percentage were used for qualitative data description, while chi-squared test and Fisher's exact test were used for their comparisons based on their expected numbers. Rates were compared using log-rank test. A *p*-value <0.050 was used as a significant cut-off point.

## 3. Results

**Figure 1:**
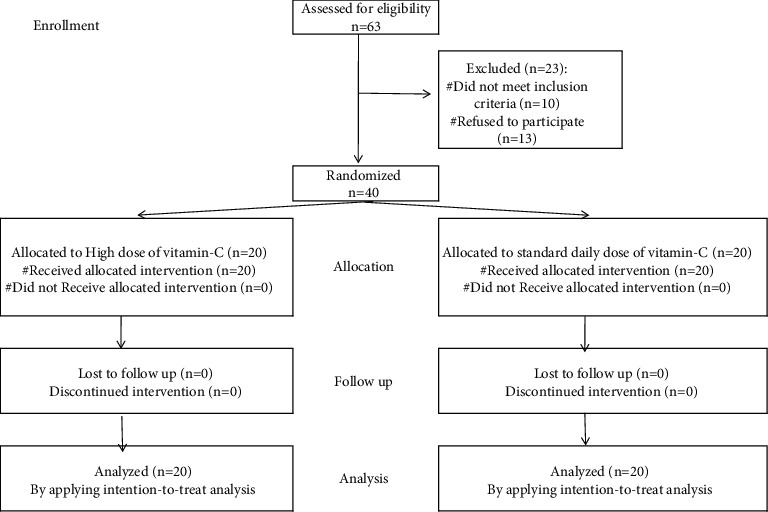
Flow diagram of the study.

Among 63 eligible patients, 40 patients were included in the study and were randomly allocated to 2 groups (20 each). 23 patients were excluded where 10 patients did not meet the inclusion criteria and 13 patients declined to participate (Figure 1). The baseline demographics and clinical and laboratory characteristics were comparable between the study groups (Tables 1 and 2).

The change in SOFA score (day 0–day 7) showed a significant difference between the two groups (Group I, −3.2 ± 2.8 vs. Group II, 0.8 ± 3.3, *p* < 0.001) (Table 3). The SOFA score was significantly higher at day 4 (9.0 ± 2.6 vs. 5.2 ± 2.0, *p* < 0.001) and day 7 (10.5 ± 2.7 vs. 3.9 ± 2.9, *p* < 0.001) in Group II than in Group I (Table 3).

**Figure 2:**
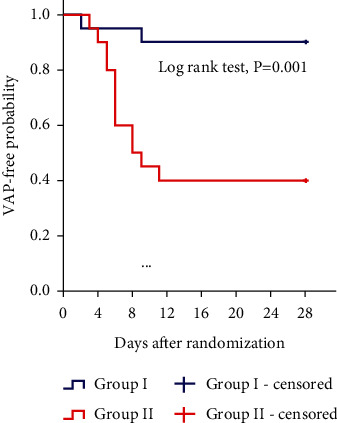
Kaplan–Meier plot estimates of the risk of ventilator-associated pneumonia (VAP) occurring in 28 days between the study groups.

The incidence of early VAP was significantly lower in Group I (5.0%) than in Group II (35.0%) (*p*=0.044), while the incidence of late VAP was comparable between the study groups (Table 3). The time to occurrence of VAP indicated that the proportion of patients without pneumonia was higher in Group I than in Group II (log-rank test, *p*=0.001) (Figure 2).

**Figure 3:**
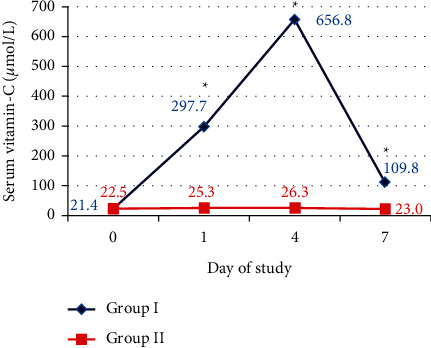
Serum vitamin C levels between the study groups. ^*∗*^Significant.

The baseline plasma vitamin C levels were deficient <50–70 *µ*mol/L in both groups [24]. Vitamin C levels showed a significant rise in Group I more than Group II at day 1 and day 4 (*p* < 0.001). Despite the decrease of vitamin C levels at day 7 in both groups, vitamin C levels were significantly higher in Group I than in Group II (*p* < 0.001) (Table 3) (Figure 3).

In Group I, the serum GPX activity of day 4 and day 7 was significantly higher (*p*=0.005, *p*=0.014), whereas CRP levels of day 4 and day 7 were significantly lower than Group II (*p* < 0.001, *p* < 0.001) (Table 3).

**Figure 4:**
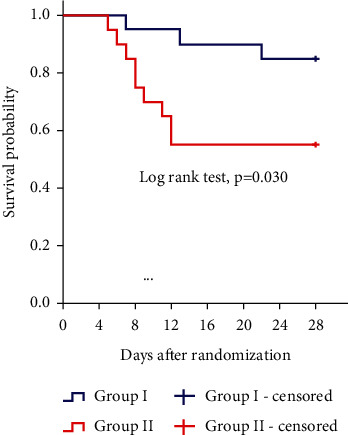
Kaplan–Meier plot for 28-day mortality.

The duration of vasopressor therapy was significantly shorter in Group I than in Group II (55.8 ± 21.6 vs. 75.6 ± 33.3 hours, *p*=0.033) (Table 3). Over and above, in Group II, the incidence of 28-day mortality was significantly higher than Group I (45% vs. 15%, *p*=0.038) (Table 3), and the Kaplan–Meier plot for 28-day mortality showed that the risk of mortality was significantly reduced in Group I in comparison to Group II (log-rank test, *p*=0.030) (Figure 4).

VFDs and ICU length of stay were comparable between the study groups (Table 3).

Patients of both groups suffered from some tolerable side effects such as vomiting, hematuria, hypernatremia ,and flushing which did not result in vitamin C cessation or withdrawal from the study. The incidences of all adverse events were comparable between the study groups.

## 4. Discussion

This study demonstrated that the high-dose vitamin C IVI in patients with sepsis requiring MV was associated with improvement in the organ dysfunction through statistically significant reduction in SOFA score and decreased severity of illness in the Group I compared to Group II. Also, there was a statistically significant reduction in the incidence of early VAP and CRP levels in the Group I in comparison to Group II. In addition, plasma vitamin C levels and GPX activity were statistically significantly higher in the Group I than in Group II. Moreover, there was a statistically significant reduction in 28-day mortality and duration of vasopressor therapy in the Group I compared to Group II. However, there were no statistically significant differences in the VFDs and ICU length of stay between the study groups.

The high-dose vitamin C IVI was found as adjuvant treatment in sepsis due to its antioxidant, anti-inflammatory, and immune-supportive roles [7, 25, 26]. Fowler et al. demonstrated that the high-dose vitamin C IVI reduced the organ failure accompanied by sepsis in a dose-dependent manner. Also, they reported that the IV doses can achieve plasma concentrations 30- to 70-fold higher than the maximum accepted oral doses [22]. Moreover, Carr et al. documented that parenteral intake was better than oral route in critically ill patients to get the therapeutic plasma vitamin C levels necessary to prevent oxidative stress and modulate immune responses (OS) [8]. Therefore, the research team chose the IVI route to achieve high plasma vitamin C levels.

The high-dose vitamin C IVI (1.5 g/6 h) for 4 consecutive days was selected based on several safety and efficacy studies which reported that the high-dose vitamin C IVI was safe and well-tolerated and may have positive impacts on the extent of multiple organ failure [8, 13, 22, 24].

In this study, baseline vitamin C levels were deficient in Group I and Group II (21.4 ± 10.6 and 22.5 ± 12.3 *µ*mol/L, respectively), while the normal human vitamin C levels are 50–70 *µ*mol/L [24]. These results were in accordance with Fowler et al., who reported that severe deficiency in plasma vitamin C levels was found in critically ill patients with less than 27 *µ*mol/L [22]. A previous study reported that septic patients require up to 3000 mg/day of vitamin C to normalize their plasma level (68 *µ*mol/L) [13]. The presence of significant high levels of vitamin C on day 1, day 4, and day 7 in Group I in comparison to Group II were in agreement with Fowler et al., who documented that the administration of high-dose vitamin C IVI (50 mg/kg/24 hours and 200 mg/kg/24 hours) every 6 hours produced sustained steady-state plasma levels of vitamin C [22].

In critically ill patients, SOFA scores are strong indicators of overall mortality and CRP levels are robust indicators for extent of infection, incidences of organ injury and death [22]. The results of the current study showed that both SOFA score and CRP level at day 4 and day 7 were significantly reduced in Group I patients in comparison to Group II patients. In addition, the duration of vasopressor therapy and the 28-day mortality were significantly lower in Group I than in Group II. Many preclinical studies confirmed that vitamin C had beneficial effects on improving the survival and outcomes of critically ill patients with sepsis and septic mouse models [7, 25, 26].

Consistent with our results, Lv et al. reported that vitamin C has an organ protective effect in septic patients proved by significant differences in the change of SOFA score and 28-day mortality, after early treatment with high-dose vitamin C IVI in combination with the standard treatment for sepsis, in favor of the intervention group over the control group [27].

The results of this study were in accordance with Fowler et al., who found an effective reduction of SOFA score and CRP level over 96 hours in the intervention group in comparison to placebo group. However, they reported a nonsignificant difference in 28-day mortality among study groups, and this could be attributed to the small sample size in their study that included only 8 patients in each group [22].

Furthermore, results of this research matched with Scholz et al., who conducted a systematic review and meta-analysis study which showed that vitamin C administration for 3 to 4 days reduced the short-term mortality (< 30 days) in patients with sepsis [28]. Moreover, a retrospective cohort study conducted by Gao et al. reported that the high-dose vitamin C (6 g/12 h on the first day then 6 g once daily for the following 4 days) in COVID-19 patients could lower the mortality with reduced CRP levels in the high-dose vitamin C group compared to the standard therapy group [9]. Over and above, Wilson et al. documented that parenteral intake of high-dose vitamin C as an adjuvant therapy for sepsis could attenuate the progress of sepsis and improve survival [24].

In agreement with the current study results, Zabet et al. demonstrated that the use of high-dose vitamin C was associated with a significant decrease in the dose and duration of norepinephrine infusion and also a marked reduction in 28-day mortality in septic shock patients [13]. Moreover, Marik et al. documented that the initial use of IV vitamin C therapy along with hydrocortisone and thiamine could attenuate organ dysfunction and reduce the hospital mortality [20].

The investigators of this clinical trial recorded that the administration of high-dose vitamin C IVI lowered the incidence of early VAP in the Group I in comparison to Group II. These results were supported by Fisher et al., who reported that vitamin C prevented sepsis-induced cytokine storms that induced lung injury. Vitamin C could prevent neutrophil accumulation, increase alveolar fluid clearance, and limit lung barrier damage [7, 25]. In addition, Manzanares et al. reported that the high-dose selenium infusion with its antioxidant properties decreased the incidence of early VAP in patients with SIRS in ICU [18].

Vitamin C reduces free radicals by its antioxidant effects, enhances the immune response, and accelerates detoxification of liver enzymes [29]. Therefore, the investigators chose to incorporate vitamin C in the treatment plan of patients with sepsis requiring MV as a new strategy in the prevention of VAP. The current study reported that the GPX activity was significantly higher in Group I in comparison to Group II. These data were supported by Zal et al., who reported that the daily oral supplementation of vitamin C increased the level of GPX that scavenges free radicals and prevents oxidative damage [11]. Moreover, these results were in concordance with the data reported by Mahmoudabadi et al., who documented that vitamin C significantly improved the overall oxidative stress status including the GPX activity [30]. In addition, Hermsdorff et al. reported that GPX activity was significantly increased in healthy young adults with high vitamin C intake [31]. Consistent with our results, Manzanares et al. found that the high-dose selenium infusion with its antioxidant properties was effective in maximizing serum GPX activity [18].

Acute respiratory distress syndrome (ARDS) and VAP continue to plague mechanically ventilated patients. It is rare to discover the diffuse alveolar damage (DAD) in the absence of histological signs of pneumonia [32]. The research team proved that the high-dose vitamin C infusion was safe, decreased inflammation, enhanced recovery to infections, and improved health status.

In this study, the ICU length of stay was comparable between the study groups. In concordance with our results, Marik et al. found that there was no significant difference in length of ICU stay in patients with severe sepsis or septic shock who had IV vitamin C therapy along with hydrocortisone and thiamine [20].

This study proved that the high-dose vitamin C was tolerable, safe, and did not result in any patient withdrawal from the study. In accordance with the results of this study, Fowler et al. and Hoffer et al. reported that the administration of various vitamin C doses did not result in any serious adverse events [22, 33].

In contrast to results obtained in this study, Fowler et al. showed that 96-hour infusion of vitamin C had no significant difference in modified SOFA score or VFDs between the study groups. However, there were higher ICU-free days till 28-day, higher hospital-free days till 60-day, and lower 28-day mortality rate in the vitamin C group. These differences might be explained by the advanced stages of sepsis that were found before the ARDS development, the delay and insufficient dose of vitamin C administration in the study group, and heterogeneity of patients' baseline characteristics [17].

Also, Litwak et al. reported that there were no decreases in ICU length of stay, hospital length of stay, the duration of vasopressor therapy, and mortality in patients with septic shock receiving IV vitamin C therapy along with hydrocortisone and thiamine [34]. Different results of Litwak et al. could be attributed to: First, insufficient duration of therapy due to patient's death or discontinuation by the physicians before 4 days or until ICU discharge. So, this might have decreased the benefits of treatment in the study group. Second, the initiation or discontinuation of therapy depended on the preference of physicians, which might represent a confounding factor. Third, the insufficient lab values for SOFA score calculation resulted in the inaccurate assessment of SOFA score changes. Fourth, an inappropriate selection of empiric antimicrobial therapy was observed. Fifth, the absence of a high-dose vitamin C only arm was noticed. Sixth, the therapy was used as a last-line treatment in patients who were not clinically improved which might have limited efficacy in that study [34].

Ahn et al. found that patients in high-dose vitamin C IVI group had nonsignificant differences in the changes of SOFA score, time to shock reversal, and 90-day mortality in comparison to the control group [35]. This could be attributed to selection bias, clinical practice variations between physicians, and not measuring the plasma levels of vitamin C.

Moreover, the results of this study differed from Hemilä et al. who conducted a meta-analysis study which showed that vitamin C decreased the ventilation duration in critically ill patients explained by the variation of the severity of the medical conditions in the included studies which required different ventilation durations [36].

This study had several limitations. First, larger sample size is required in the near future to verify the obtained significance in this study. Second, this study was carried out in a single center. So, further multicenter trials are required. Third, the research team did not assess the corticosteroid use with the high-dose vitamin C IVI. Corticosteroid use might interfere with the results of this study, decreased the vasopressor dose, increased the incidence of HAP, and, hence, increased MV duration and overall mortality as shown in previous studies [37, 38]. Fourth, the majority of results of this trial were evaluated at day 7 and not at day 14 or day 28 because 14 or 28 days would be a relatively long duration with respect to the limited healthcare resources for patients' follow-up with the risk of missing data. Fifth, for economic implications, the hospital length of stay should be examined in further randomized clinical trials, as it is an important outcome. Sixth, the researchers measured the mean SOFA score at a defined time point in the study not the mean SOFA respiratory score, which evaluates the degree of respiratory dysfunction and the effects of pulmonary infections. However, the investigators preferred measuring the degree of illness severity by using the change in the SOFA score from baseline/maximum to a defined time point, which is a good indicator for predicting overall mortality [39]. Seventh, the research team did not assess the proinflammatory cytokines. A validated assay of proinflammatory cytokines will provide critical information to ICU teams to mitigate the negative effects of inflammation.

**Figure 5:**
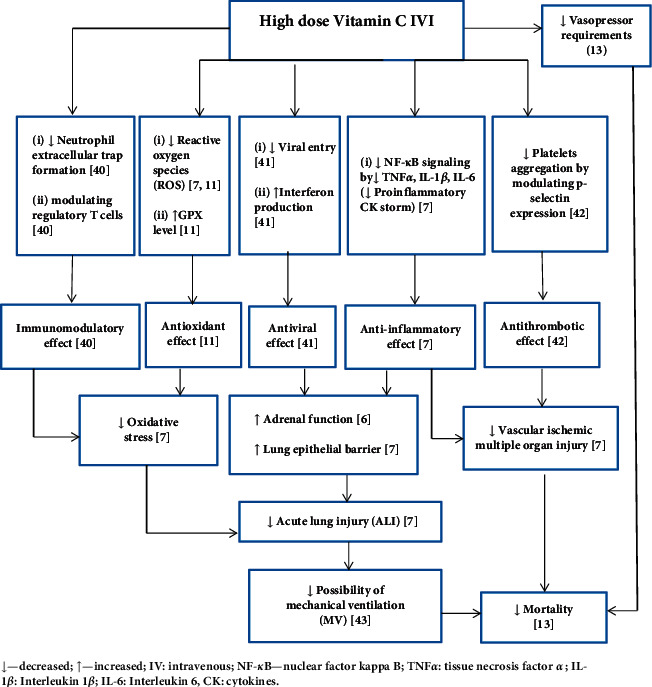
Possible roles of high-dose vitamin C intravenous infusion (IVI) in targeting the pathophysiology of sepsis requiring mechanical ventilation.

Possible roles of high-dose vitamin C IVI in targeting the pathophysiology of sepsis requiring MV are presented in Figure 5 [6, 7, 11, 13, 40–43].

Recently, at that time of COVID-19, the results from this study served as a logical clue to support the early administration of high-dose vitamin C IVI. This useful, inexpensive, and readily available intervention with no obvious side effects might be an attractive adjunctive therapy for the treatment of patients with sepsis requiring MV.

## 5. Conclusion

The early use of high-dose vitamin C intravenous infusion in patients with sepsis requiring mechanical ventilation in combination with the standard treatment for sepsis significantly improved illness severity, increased antioxidant status, decreased inflammation, reduced incidence of ventilator-associated pneumonia, and reduced mortality with no obvious adverse events.

## Figures and Tables

**Table 1:** Baseline demographic and clinical characteristics between the study groups.

Variables		Group I (*n* = 20)	Group II (*n* = 20)	*p* value
Age (years)		53.0 ± 23.3	52.1 ± 18.8	^0.900
Sex (*n*, %)	Male	9 (45.0%)	12 (60.0%)	#0.342
	Female	11 (55.0%)	8 (40.0%)	
Body mass index (BMI) (kg/m^2^)		28.2 ± 5.9	28.7 ± 6.3	^0.810
APACHE II		23.2 ± 4.7	22.6 ± 5.4	**^**0.712
Time from ICU admission up to the diagnosis of sepsis (hours)		13.5 ± 3.1	13.4 ± 3.5	**^**0.888

*Physiological variables at day 0 (randomization)*				
White blood cell count (WBCs) (x10^3^/mL)		17.4 ± 6.5	17.0 ± 6.9	^0.855
Serum creatinine (mg/dL)		0.80 ± 0.27	0.90 ± 0.42	^0.380
Lactate (mmol/L)		3.6 ± 0.4	3.7 ± 0.5	^0.405
PaO_2_/FiO_2_ ratio		195.7 ± 98.8	201.1 ± 128.2	^0.883

Data were presented as mean and standard deviation or number and (%). APACHE: Acute Physiology and Chronic Health Evaluation; FiO_2_: fraction of inspired oxygen; PaO_2_: partial pressure of oxygen; SD: standard deviation; SOFA: sepsis-related organ failure assessment. ^Independent *t*-test, ^#^chi-squared test, ^§^Fisher's exact test.

**Table 2:** Comorbidities, diagnosis, and source of sepsis between the study groups.

Variables		Group I (*n* = 20)	Group II (*n* = 20)	*p* value
Comorbidities (*n*, %)	Hypertension	11 (55.0%)	10 (50.0%)	#0.752
	Diabetes mellitus	7 (35.0%)	6 (30.0%)	#0.736
	Chronic liver disease	6 (30.0%)	4 (20.0%)	#0.465
	Deep vein thrombosis	4 (20.0%)	2 (10.0%)	§0.661
	Ischemic heart disease	4 (20.0%)	5 (25.0%)	§1.000
	Stroke	3 (15.0%)	4 (20.0%)	§1.000
	Autoimmune disease	1 (5.0%)	3 (15.0%)	§0.605

Diagnosis (n, %)	Cirrhosis	3 (15.0%)	5 (25.0%)	§0.275
	Trauma	6 (30.0%)	1 (5.0%)	
	Bed sores	4 (20.0%)	2 (10.0%)	
	Bowel perforation	4 (20.0%)	5 (25.0%)	
	Acidosis	1 (5.0%)	2 (10.0%)	
	Unknown	2 (10.0%)	5 (25.0%)	

Source of sepsis (n, %)	Central venous catheter	7 (35.0%)	6 (30.0%)	§0.913
	Urinary tract infection	5 (25.0%)	6 (30.0%)	
	Abdominal	4 (20.0%)	2 (10.0%)	
	Skin and soft tissue	2 (10.0%)	4 (20.0%)	
	Infected diabetic foot	2 (10.0%)	2 (10.0%)	

Data were presented as number and (%). #Chi-squared test. §Fisher's exact test.

**Table 3:** Clinical outcomes between the study groups.

Variables	Group I (*n* = 20)	Group II (*n* = 20)	*p* value
Early VAP, number of episodes (%)	1(5.0%)	7(35.0%)	§0.044^*∗*^
Late VAP, number of episodes (%)	1(5.0%)	5(25%)	**⌂**0.182
SOFA score, mean ± SD			
SOFA score (day 0)	12.1 ± 1.6	12.7 ± 2.1	^⋀^0.354
SOFA score (day 1)	8.8 ± 1.4	9.8 ± 1.8	^⋀^0.057
SOFA score (day 4)	5.2 ± 2.0	9.0 ± 2.6	^⋀^<0.001^*∗*^
SOFA score (day 7)	3.9 ± 2.9	10.5 ± 2.7	^⋀^<0.001^*∗*^
DSOFA (day 0–day 7)	−3.2 ± 2.8	0.8 ± 3.3	^⋀^<0.001^*∗*^
Plasma vitamin C level, mean ± SD			
Plasma vitamin C level (day 0) (µmol/L)	21.4 ± 10.6	22.5 ± 12.3	^⋀^0.759
Plasma vitamin C level (day 1) (µmol/L)	297.7 ± 92.6	25.3 ± 12.4	^⋀^<0.001^*∗*^
Plasma vitamin C level (day 4) (µmol/L)	656.8 ± 125.6	26.3 ± 10.8	^⋀^<0.001^*∗*^
Plasma vitamin C level (day 7) (µmol/L)	109.8 ± 28.7	23.0 ± 10.8	^⋀^<0.001^*∗*^
GPX activity, Mean ± SD			
GPX (day 0) (U/ml)	0.308 ± 0.172	0.327 ± 0.206	^⋀^0.760
GPX (day 1) (U/ml)	0.498 ± 0.174	0.387 ± 0.203	^⋀^0.071
GPX (day 4) (U/ml)	0.648 ± 0.295	0.408 ± 0.209	^⋀^0.005^*∗*^
GPX (day 7) (U/ml)	0.481 ± 0.292	0.275 ± 0.209	^⋀^0.014^*∗*^
CRP levels, mean ± SD			
CRP (day 0) (mg/L)	253.3 ± 47.2	255.9 ± 48.5	^⋀^0.864
CRP (day 1) (mg/L)	232.0 ± 47.0	261.6 ± 48.4	^⋀^0.057
CRP (day 4) (mg/L)	175.9 ± 57.6	307.5 ± 74.4	^⋀^<0.001^*∗*^
CRP (day 7) (mg/L)	168.0 ± 57.4	314.7 ± 74.8	^⋀^<0.001^*∗*^
Mean of vasopressor therapy duration (hours)	55.8 ± 21.6	75.6 ± 33.3	^⋀^0.033^*∗*^
28-day mortality, n, (%)	3 (15.0%)	9 (45%)	**⌂**0.038
Ventilator-free days (days), median (IQR)	22 (21.0–23.0)	21.5 (20.3–22.0)	#0.084
ICU LOS (days), median (IQR)	9.0 (8.0–11.0)	13.5 (8.0–24.8)	#0.081

Data were presented as mean and standard deviation (SD), number and percentage or median, 1^st^& 3^rd^ interquartile range. ^*∗*^Significant. CRP: C-reactive protein; DSOFA: delta SOFA (change in SOFA score) (negative values indicate reduction); GPX: glutathione peroxidase; ICU: intensive care unit; ICU LOS: ICU length of stay; VAP: ventilator-associated pneumonia, ^independent *t*-test, **⌂**chi-squared test, #Mann–Whitney *U* test, §Fisher's exact test.

## Data Availability

The datasets generated and analyzed in the current study are available from the corresponding author on reasonable request.

## Abbreviations

APACHE II: Acute Physiology and Chronic Health Evaluation
ARDS: Acute respiratory distress syndrome
CRP: C-reactive protein
DAD: Diffuse alveolar damage
GPX: Glutathione peroxidase activity
HAP: Hospital-acquired pneumonia
ICU: Intensive care unit
IVI: Intravenous infusion
MV: Mechanical ventilation
PaO_2_/FiO_2_: Ratio of arterial oxygen partial pressure to fractional inspired oxygen
SOFA: Sequential organ failure assessment
VAP: Ventilator-associated pneumonia
VFDs: Ventilator-free days.
